# Supplementary material for: Identification of a peptide motif that potently inhibits two functionally distinct subunits of Shiga toxin
Source: Commun Biol. 2021 May 10;4:538. doi: 10.1038/s42003-021-02068-3 (PMC8111002; doi:10.1038/s42003-021-02068-3)
Supplement: Supplementary file 2 — Supplementary Information [file 42003_2021_2068_MOESM2_ESM.pdf]

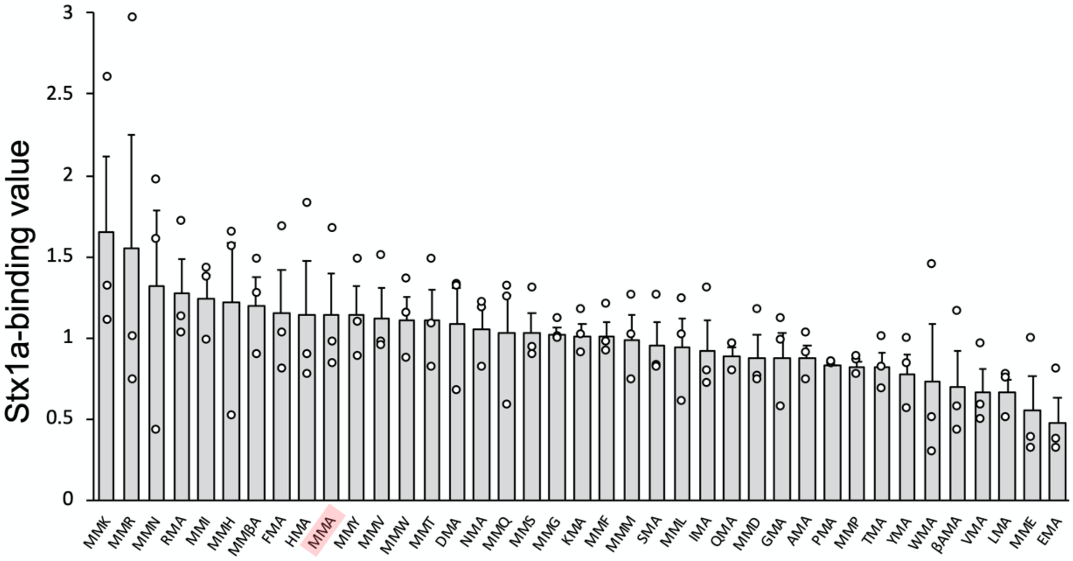

Supplementary Fig. 1 Identification of high-affinity, Stx1a-binding motifs by screening tetravalent peptides synthesized on a cellulose membrane.

The sum of the pixel values of all peptide spots (Fig. 1a) was normalized to 40 (i.e., the number of tetravalent peptides synthesized on the membrane), so that each peptide would have a value 1 in the absence of selectivity for Stx1a. The data are presented as a normalized binding value (mean ± SE, *n*=3).

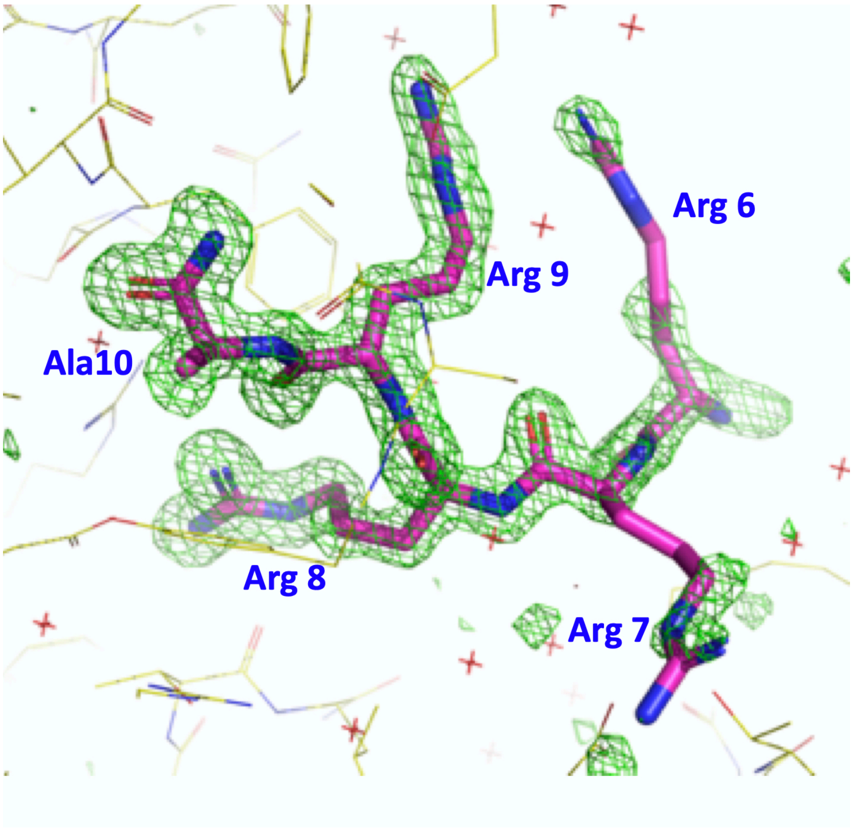

Supplementary Fig. 2 Simulated annealing mFo-DFc omit map for bound peptide in Stx2a. The positive mFo-DFc electron density of the peptide was contoured at 3 sigma. MMβA-mono is shown in magenta.

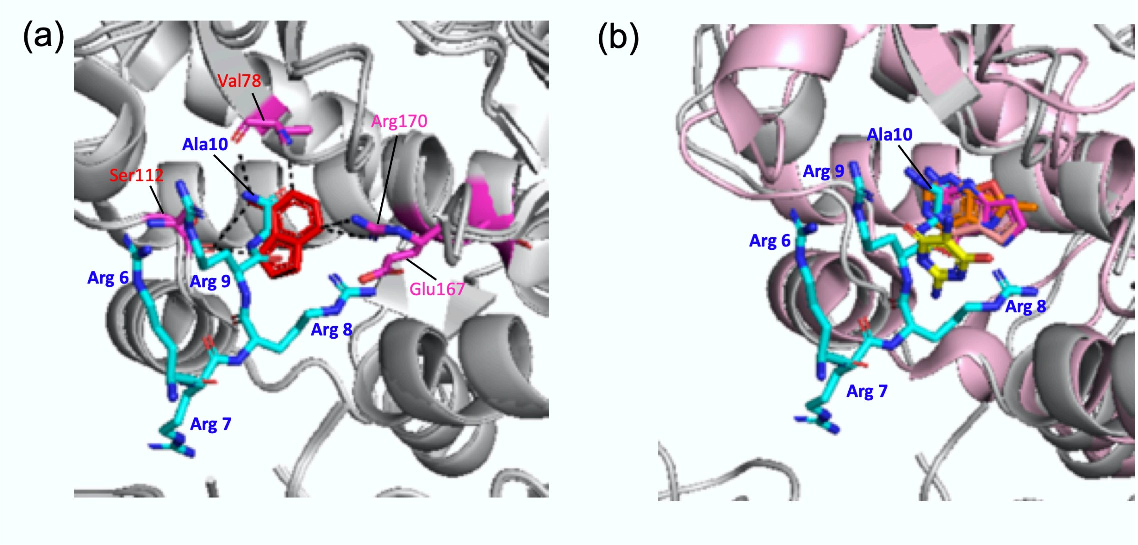

Supplementary Fig. 3 MMβA-mono occupied the wider region of the catalytic region of the Stx2a A-subunit, compared to previously developed inhibitory compounds[15]. The catalytic region of the Stx2a A-subunit (gray) in complex with MM*β* A-mono (light blue) or adenine (red) (a), and that of ricin (light pink), whose catalytic region is highly similar to the A-subunit, in complex with small inhibitory compounds (magenta, orange, pink, and yellow) (b) were superimposed. Magenta, orange, pink, and yellow indicate 7-deazaguanine (PDB ID: 1il3), 9-deazaguanine (PDB ID: 1il4), 2,5-diaminnno-4,6-dihydroxypyrimidine (PDB ID: 1il5), and 5-amino-2-methyl-6h-oxazolo[5,4-D]pyrimidin-7-one (PDB ID: 1il9), respectively.

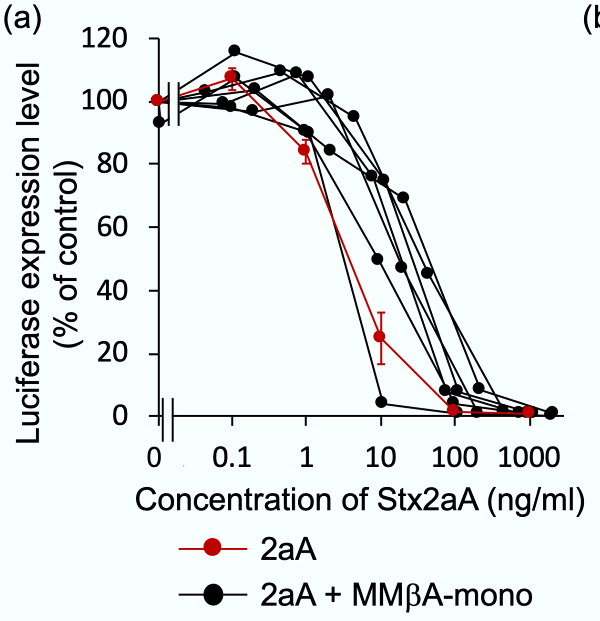

(b)

	IC50 value mean (ng/ml) ± SE
2aA	10.0 ± 3.8
2aA + MMβA-mono	26.5* ± 10

Supplementary Fig. 4 Inhibitory effect of MMβA-mono on the catalytic activity of the Stx2a A-subunit.

The inhibitory effect of MMβA-mono on the catalytic activity of the Stx2a A-subunit was examined using the TNT Quick Coupled Translation System, in which newly synthesized luciferase expression levels can be monitored in a reticulocyte lysate system (Promega Corp., WI, USA). Biotinylated MMβA-mono (70 μg/ml) was incubated with streptavidin beads for 2 h at 4°C. After washing, the beads were incubated with the Stx2a A-subunit (75 μg/ml) overnight at 4°C to form a complex of the Stx2a A-subunit and MMβA-mono. Various amounts of purified Stx2a A-subunit (n = 7) or Stx2a A-subunit complexed with MMβA-mono (n = 7) were used to measure the activity of the A-subunit in the TNT system. Data are presented as a percentage of the control value, which represents the luciferase expression level synthesized in the absence of the Stx2a A-subunit in the translation assay (a). The concentration of the Stx2a A-subunit that demonstrated 50% inhibition was determined as the IC50 value (mean ± SE, n = 7) (b).
* $P < 0.05$ by Student's t-test.

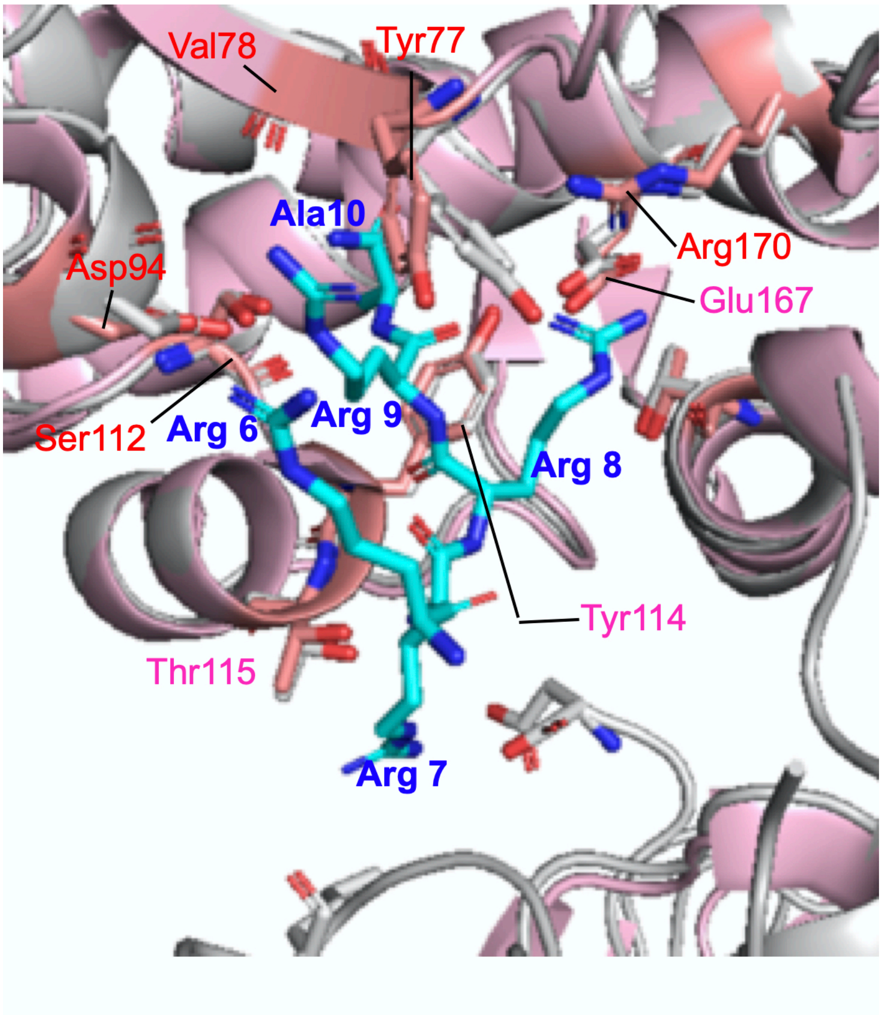

Supplementary Fig. 5 MMβA-mono can bind to the Stx1a A-subunit with highly similar interactions as those to the Stx2a A-subunit.

The catalytic region of Stx2a A-subunit (gray) in complex with MMβA-mono (light blue) and the catalytic region of Stx1a A-subunit (light pink; PDB ID: 1dm0) were superimposed.
