# Supplementary material for: Identification of a peptide motif that potently inhibits two functionally distinct subunits of Shiga toxin
Source: Commun Biol. 2021 May 10;4:538. doi: 10.1038/s42003-021-02068-3 (PMC8111002; doi:10.1038/s42003-021-02068-3)
Supplement: Supplementary file 3 — Description of Additional Supplementary Files [file 42003_2021_2068_MOESM3_ESM.pdf]

Description of Additional Supplementary Files

File Name: Supplementary Data 1

Description: Source data underlying plots shown in figures.
