# Supplementary material for: Identification of a peptide motif that potently inhibits two functionally distinct subunits of Shiga toxin
Source: Commun Biol. 2021 May 10;4:538. doi: 10.1038/s42003-021-02068-3 (PMC8111002; doi:10.1038/s42003-021-02068-3)
Supplement: Supplementary file 5 — Reporting Summary [file 42003_2021_2068_MOESM5_ESM.pdf]

nature research

Corresponding author(s): Kiyotaka Nishikawa

Last updated by author(s): 2021-3-17

Reporting Summary

Nature Research wishes to improve the reproducibility of the work that we publish. This form provides structure for consistency and transparency in reporting. For further information on Nature Research policies, see our Editorial Policies and the Editorial Policy Checklist.

Statistics

For all statistical analyses, confirm that the following items are present in the figure legend, table legend, main text, or Methods section.

n/a	Confirmed	
☐	☒	The exact sample size (*n*) for each experimental group/condition, given as a discrete number and unit of measurement
☐	☒	A statement on whether measurements were taken from distinct samples or whether the same sample was measured repeatedly
☐	☒	The statistical test(s) used AND whether they are one- or two-sided<br>*Only common tests should be described solely by name; describe more complex techniques in the Methods section.*
☒	☐	A description of all covariates tested
☐	☒	A description of any assumptions or corrections, such as tests of normality and adjustment for multiple comparisons
☐	☒	A full description of the statistical parameters including central tendency (e.g. means) or other basic estimates (e.g. regression coefficient) AND variation (e.g. standard deviation) or associated estimates of uncertainty (e.g. confidence intervals)
☐	☒	For null hypothesis testing, the test statistic (e.g. *F*, *t*, *r*) with confidence intervals, effect sizes, degrees of freedom and *P* value noted<br>*Give P values as exact values whenever suitable.*
☒	☐	For Bayesian analysis, information on the choice of priors and Markov chain Monte Carlo settings
☒	☐	For hierarchical and complex designs, identification of the appropriate level for tests and full reporting of outcomes
☒	☐	Estimates of effect sizes (e.g. Cohen's *d*, Pearson's *r*), indicating how they were calculated

Our web collection on statistics for biologists contains articles on many of the points above.

Software and code

Policy information about availability of computer code

Data collection	Data process: XDS & XSCALE (version Jan 26, 2018)
Data analysis	Crystallographic refinement : Phenix 1.14_3260, Modeling : Coot 0.9, Graphics: Pymol2.3.2, Statistical analysis : SPSS Statistics ver.27

For manuscripts utilizing custom algorithms or software that are central to the research but not yet described in published literature, software must be made available to editors and reviewers. We strongly encourage code deposition in a community repository (e.g. GitHub). See the Nature Research guidelines for submitting code & software for further information.

Data

Policy information about availability of data

All manuscripts must include a data availability statement. This statement should provide the following information, where applicable:
- Accession codes, unique identifiers, or web links for publicly available datasets
- A list of figures that have associated raw data
- A description of any restrictions on data availability

The crystal structures have been deposited in the Protein Data Bank (PDB) with the following accession no. 7D6Q and 7D6R. All data are available from corresponding author upon reasonable request.

Field-specific reporting

Life sciences study design

All studies must disclose on these points even when the disclosure is negative.

Sample size	No statistical methods were used to determine the sample size. We repeated each experiment at least three times and results were reproducibly obtained.
Data exclusions	No data were excluded.
Replication	All attempts at replication were successful.
Randomization	N/A
Blinding	N/A

Reporting for specific materials, systems and methods

We require information from authors about some types of materials, experimental systems and methods used in many studies. Here, indicate whether each material, system or method listed is relevant to your study. If you are not sure if a list item applies to your research, read the appropriate section before selecting a response.

Materials & experimental systems

n/a	Involved in the study
☐	☒ Antibodies
☐	☒ Eukaryotic cell lines
☒	☐ Palaeontology and archaeology
☒	☐ Animals and other organisms
☒	☐ Human research participants
☒	☐ Clinical data
☒	☐ Dual use research of concern

Methods

n/a	Involved in the study
☒	☐ ChIP-seq
☒	☐ Flow cytometry
☒	☐ MRI-based neuroimaging

Antibodies

Antibodies used	Anti-His-tag antibody (clone: 9C11, Wako Pure industries, Japan), Anti-Stx 1aA-subunit specific monoclonal antibody (originally obtained), Anti-Stx 2aA-subunit specific monoclonal antibody (originally obtained).
Validation	Anti-His-tag antibody are commonly available and validated by the manufacture. Prior to using, we tested the reactivity of the purchased antibodies against the samples alongside the positive control ensured in the manufacture's data sheet. Anti-Stx 1aA-subunit or Stx2aA-subunit specific monoclonal antibody was validated by western blotting using recombinant Stx 1aA-subunit or Stx2aA-subunit, respectively.

Eukaryotic cell lines

Policy information about cell lines

Cell line source(s)	Vero cells (RCB0001)
Authentication	Authentication was performed by RIKEN BRC Cell Bank initially.
Mycoplasma contamination	The cell lines were negative for Mycoplasma contamination test.
Commonly misidentified lines (See ICLAC register)	No commonly misidentified cell lines were used.
